# Supplementary material for: Germline burden of rare damaging variants negatively affects human healthspan and lifespan
Source: eLife. 2020 Apr 7;9:e53449. doi: 10.7554/eLife.53449 (PMC7314550; doi:10.7554/eLife.53449)
Supplement: Transparent reporting form [file elife-53449-transrepform.docx]

***eLife’s* transparent reporting form**

We encourage authors to provide detailed information *within their submission* to facilitate the interpretation and replication of experiments. Authors can upload supporting documentation to indicate the use of appropriate reporting guidelines for health-related research (see [EQUATOR Network](http://www.equator-network.org/%20)), life science research (see the [BioSharing Information Resource](https://biosharing.org/)), or the [ARRIVE guidelines](http://www.plosbiology.org/article/info:doi/10.1371/journal.pbio.1000412) for reporting work involving animal research. Where applicable, authors should refer to any relevant reporting standards documents in this form.

If you have any questions, please consult our Journal Policies and/or contact us: [editorial@elifesciences.org](mailto:editorial@elifesciences.org).

**Sample-size estimation**

- You should state whether an appropriate sample size was computed when the study was being designed
- You should state the statistical method of sample size computation and any required assumptions
- If no explicit power analysis was used, you should describe how you decided what sample (replicate) size (number) to use

Please outline where this information can be found within the submission (e.g., sections or figure legends), or explain why this information doesn’t apply to your submission:

**Replicates**

Sample size considerations were driven by the following factors:

1) Data availability. Most of exome sequencing datasets do not include participants that are followed up for a period long enough to acquire lifespan data. In this regard, the UK biobank dataset is unique in that it followed up participants and it is large enough to perform association studies. UK brain bank network (UKBBN) sequenced postmortem tissues, thus the lifespan data is available.

2) Previous studies assessing protein-truncating variant burden with various phenotypes include several thousand subjects, which approximately corresponds to lifespan analyses in our dataset (~2,400 subjects total), while our healthspan analysis significantly exceeds these sample sizes and includes ~40k participants.

3) With this sample size available, we observed significant effects in both datasets. All methods and datasets are explicitly described in Methods.

- You should report how often each experiment was performed
- You should include a definition of biological versus technical replication
- The data obtained should be provided and sufficient information should be provided to indicate the number of independent biological and/or technical replicates
- If you encountered any outliers, you should describe how these were handled
- Criteria for exclusion/inclusion of data should be clearly stated
- High-throughput sequence data should be uploaded before submission, with a private link for reviewers provided (these are available from both GEO and ArrayExpress)

Please outline where this information can be found within the submission (e.g., sections or figure legends), or explain why this information doesn’t apply to your submission:

We tested association of protein-truncating variant burden with lifespan in two independent WES datasets, which represents two biological replicates. Exclusion and inclusion criteria for both datasets could be found in the Data filtering section of Materials and Methods.

**Statistical reporting**

- Statistical analysis methods should be described and justified
- Raw data should be presented in figures whenever informative to do so (typically when N per group is less than 10)
- For each experiment, you should identify the statistical tests used, exact values of N, definitions of center, methods of multiple test correction, and dispersion and precision measures (e.g., mean, median, SD, SEM, confidence intervals; and, for the major substantive results, a measure of effect size (e.g., Pearson's r, Cohen's d)
- Report exact p-values wherever possible alongside the summary statistics and 95% confidence intervals. These should be reported for all key questions and not only when the p-value is less than 0.05.

Please outline where this information can be found within the submission (e.g., sections or figure legends), or explain why this information doesn’t apply to your submission:

(For large datasets, or papers with a very large number of statistical tests, you may upload a single table file with tests, Ns, etc., with reference to sections in the manuscript.)

Information on statistical analysis and sample sizes could be found in the caption for each figure. 95% confidence intervals for Cox proportional hazards model are reported within Results section and plotted in Figures 1, 2, 3, 5A (inset).

**Group allocation**

- Indicate how samples were allocated into experimental groups (in the case of clinical studies, please specify allocation to treatment method); if randomization was used, please also state if restricted randomization was applied
- Indicate if masking was used during group allocation, data collection and/or data analysis

Please outline where this information can be found within the submission (e.g., sections or figure legends), or explain why this information doesn’t apply to your submission:

**Additional data files (“source data”)**

Samples were allocated based on data sources: UKB and UKBBN subjects were analyzed independently.

- We encourage you to upload relevant additional data files, such as numerical data that are represented as a graph in a figure, or as a summary table
- Where provided, these should be in the most useful format, and they can be uploaded as “Source data” files linked to a main figure or table
- Include model definition files including the full list of parameters used
- Include code used for data analysis (e.g., R, MatLab)
- Avoid stating that data files are “available upon request”

Please indicate the figures or tables for which source data files have been provided:

R code used for statistical analysis will be uploaded to Github repository <https://github.com/azenin>
